# Multiparametric MRI-based radiomics for differentiating HER2-negative, HER2-low, and HER2-positive breast cancer

**DOI:** 10.3389/fmed.2026.1941968

**Published:** 2026-09-29

**Authors:** Lulu Gai, Shubin Luo, Yudong Yan, Wenzheng Lyu, Xingyu Zhao, Haiming Cao, Kaifeng Wang, Yunpeng Zhou, Qi Zhong, Fuli Zhu, Junrui Zhang, Lei Xu

**Affiliations:** 1 Department of Medical Imaging, Shengli Oilfield Central Hospital, Dongying, China; 2 Shandong Second Medical University, Weifang, China

**Keywords:** breast cancer, HER2 expression, HER2-low, machine learning, multiparametric MRI, radiomics

## Abstract

**Background:**

Breast cancer stratification increasingly extends beyond conventional TNM staging and binary human epidermal growth factor receptor 2 (HER2) classification. This shift is driven in part by the therapeutic relevance of HER2-low disease. We investigated whether multiparametric MRI-based radiomics could support pairwise discrimination among HER2-negative, HER2-low, and HER2-positive breast cancers.

**Methods:**

This retrospective single-center study included 157 patients who underwent breast MRI on two 3.0-T scanners. Radiomic features were extracted from T2-weighted, contrast-enhanced T1-weighted, and subtraction T1-weighted images. Seven classifiers were compared after a stratified 8:2 training-test split. Model development, five-fold cross-validation, and model selection were confined to the training set, and the selected logistic regression model was evaluated on the untouched test set.

**Results:**

Logistic regression had the highest mean training AUC across the three pairwise tasks (0.778) and was selected as the common final classifier. Test-set AUCs were 0.922 (95% CI, 0.750–1.000) for HER2-negative versus HER2-positive, 0.756 (95% CI, 0.529–0.938) for HER2-low versus HER2-positive, and 0.705 (95% CI, 0.493–0.893) for HER2-negative versus HER2-low classification.

**Conclusions:**

Training-set model selection followed by independent test-set evaluation identified the strongest discrimination between HER2-negative and HER2-positive tumors. Performance was less reliable when HER2-low tumors were involved, particularly for HER2-negative versus HER2-low classification. These findings are exploratory and require independent multicenter validation.

## 1 Introduction

Patients with human epidermal growth factor receptor 2 (HER2)-overexpressing breast cancer benefit substantially from HER2-targeted therapy (1–4). However, HER2 overexpression is observed in only about 20%–30% of breast tumors (5). Treatment options have historically been more limited for patients classified as having HER2-negative disease. This binary framework has been challenged by the recognition that many tumors historically grouped as HER2-negative show low but detectable HER2 expression. Antibody-drug conjugates (ADCs) have created new therapeutic opportunities for patients with low HER2 expression (HER2-low). Agostinetto et al. (6) reported differences between tumors with any HER2 expression and HER2-negative tumors in hormone receptor status, tumor growth, grade, and response to neoadjuvant chemotherapy. These findings have increased interest in more precise stratification of HER2 expression categories in breast cancer.

Beyond TNM staging, pathological biomarkers guide prognosis and treatment selection in routine breast cancer care. HER2 status is commonly classified as HER2-positive (immunohistochemistry [IHC] 3+ or IHC 2+ with fluorescence in situ hybridization [FISH] amplification), HER2-low (IHC 1+ or IHC 2+ with negative FISH), or HER2-negative (IHC 0). Trastuzumab deruxtecan, an HER2-targeted ADC, significantly improved progression-free and overall survival in patients with metastatic HER2-low breast cancer in the DESTINY-Breast04 phase 3 trial (7). Nevertheless, the imaging, molecular, and pathological distinctions among HER2-negative, HER2-low, and HER2-positive tumors remain incompletely characterized.

Single-sequence and multiparametric MRI-based radiomic models have been developed to distinguish HER2-positive from HER2-negative breast cancers. Multiparametric MRI models generally outperform single-sequence approaches, suggesting that complementary imaging information may improve the characterization of HER2-related tumor heterogeneity (8–10). However, studies specifically addressing HER2-low disease remain limited. Evidence is particularly sparse for pairwise discrimination among HER2-negative, HER2-low, and HER2-positive tumors.

In the present study, we extracted radiomic features from T2-weighted imaging (T2WI), contrast-enhanced T1-weighted imaging (CE-T1WI), and subtraction T1-weighted imaging (SUB-T1WI). We then evaluated whether these routinely available MRI sequences could support pairwise discrimination among HER2-negative, HER2-low, and HER2-positive breast cancers. Rather than proposing a definitive multiclass classifier, this study was designed as an exploratory assessment of MRI-derived biomarkers for HER2-oriented stratification.

## 2 Materials and methods

### 2.1 Study patients

This retrospective single-center study was approved by the Medical Ethics Committee of Shengli Oilfield Central Hospital. Written informed consent was waived by the ethics committe because of the retrospective nature of the study and the use of anonymized patient data. We retrospectively reviewed patients aged 20–60 years with surgically confirmed breast masses who underwent breast MRI at Shengli Oilfield Central Hospital between August 2021 and August 2023.

The overall workflow for patient selection, MRI acquisition, HER2 grouping and pairwise classification is summarized in Figure 1. A total of 204 cases were initially retrieved from the institutional Picture Archiving and Communication System (PACS). Cases were excluded because of incomplete or suboptimal imaging data, incomplete pathological information, relevant comorbid conditions, lesion diameter less than 5 mm, or absence of contrast enhancement. After applying these criteria, 157 patients were included in the final analysis. The final cohort comprised 43 patients with HER2-negative tumors, 80 with HER2-low tumors, and 34 with HER2-positive tumors.

**Figure 1:**
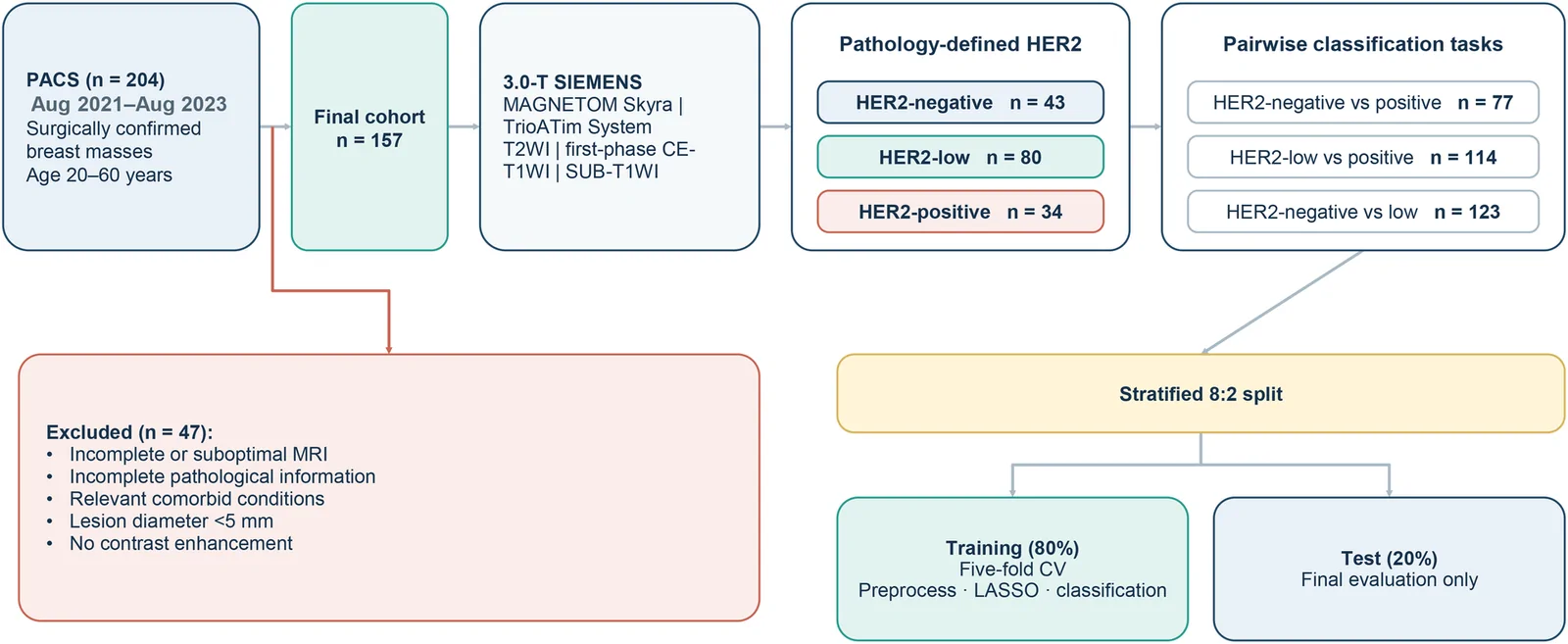
Patient selection and analysis workflow. Of 204 cases screened, 157 met the eligibility criteria, including 43 with HER2-negative, 80 with HER2-low, and 34 with HER2-positive tumors. Each pairwise dataset underwent a stratified 8:2 training-test split; model development and selection were restricted to the training set, and the test set was reserved for final evaluation.

### 2.2 MRI acquisition

All patients underwent breast MRI in the prone position using dedicated breast coils on two 3.0-T scanners. The imaging protocol included fat-suppressed T2WI, unenhanced T1-weighted imaging, diffusion-weighted imaging, and multiphase dynamic CE-T1WI. Detailed sequence parameters are provided in Table 1. Gadoteric acid meglumine was administered intravenously at a dose of 15 mL and an injection rate of 2 mL/s.

**Table 1:** MRI scanners and scan parameters.

Scanner	Sequence	TR (ms)	TE (ms)	FOV (${\mathrm{mm}}^{2}$)	Slice	Interslice
					thickness (mm)	gap (mm)
SIEMENS MAGNETOM Skyra	T2WI	6,500	81	360×360	4	1
	CE-T1WI	4.49	1.68	340×340	1.2	0.24
SIEMENS TrioATim System	T2WI	5,000	61	340×340	4	0.8
	CE-T1WI	4.23	1.57	340×340	0.9	0.18

TR, repetition time; TE, echo time; FOV, field of view.

HER2 status was categorized according to IHC and FISH results. HER2-negative tumors were defined as IHC 0. HER2-low tumors were defined as IHC 1+ or IHC 2+ with negative FISH. HER2-positive tumors were defined as IHC 3+ or IHC 2+ with positive FISH. For the radiomics analysis, T2WI, first-phase CE-T1WI, and SUB-T1WI were used. SUB-T1WI was generated by subtracting the precontrast T1WI from the corresponding first-phase CE-T1WI. The same post-contrast phase was therefore used consistently across all cases.

### 2.3 Annotation of regions of interest

Manual segmentation was performed using ITK-SNAP. As shown in Figure 2, only the largest lesion was included for patients with multiple or bilateral malignant lesions. Initial delineation was performed by eight radiologists with approximately five years of diagnostic imaging experience. All segmentations were subsequently reviewed by two senior radiologists with more than ten years of experience. The radiologists performing and reviewing the segmentations were blinded to HER2 status and other pathological findings.

**Figure 2:**
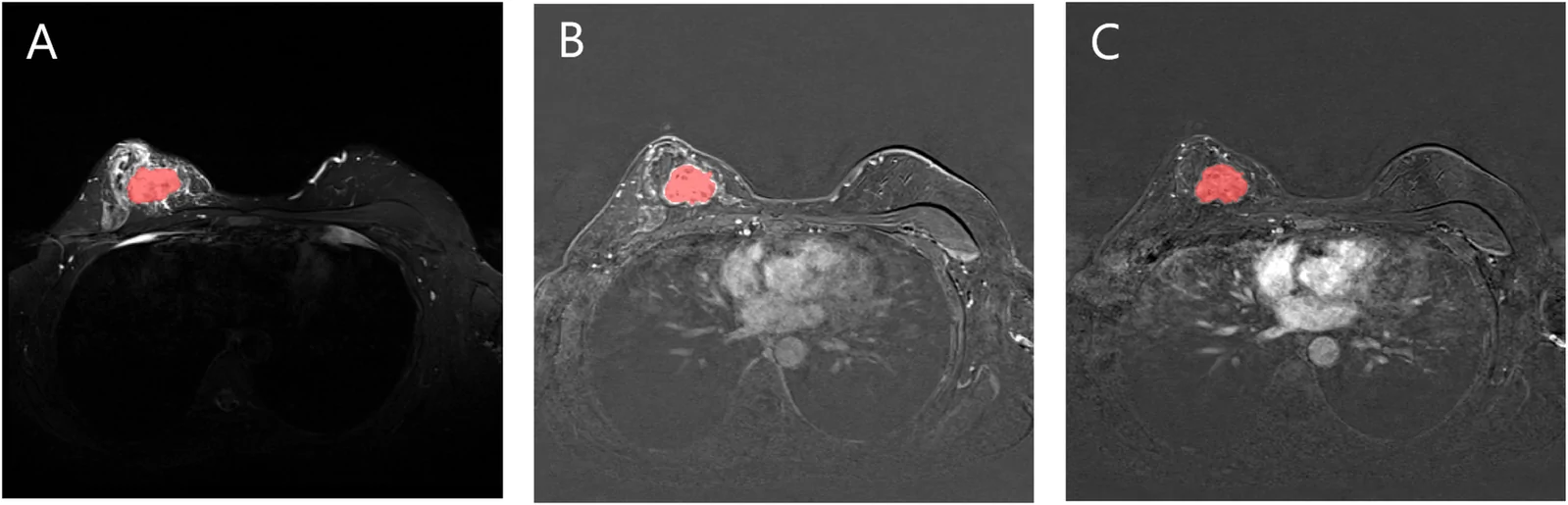
Representative multiparametric MRIs from a patient with breast cancer. The red overlay indicates the manually segmented lesion. **(A)** Axial T2-weighted image showing a right-breast lesion. **(B)** Axial first-phase contrast-enhanced T1-weighted image acquired 20 s after gadoteric acid meglumine injection. **(C)** Axial subtraction T1-weighted image generated by subtracting the unenhanced T1-weighted image from the first-phase post-contrast image shown in **(B)**.

Segmentation reproducibility was assessed using intraclass correlation coefficients (ICCs) calculated for the extracted radiomic features (11). Thirty cases were randomly selected to evaluate intra- and inter-observer agreement. For intra-observer assessment, one radiologist repeated the segmentation after a two-week interval. For inter-observer assessment, a second radiologist independently segmented the same cases. Only radiomic features with both intra- and inter-observer ICC values greater than 0.75 were retained for subsequent analyses.

### 2.4 Radiomic feature extraction

Before feature extraction, N4 bias field correction was applied to reduce intensity nonuniformity across MRI scans (12). Radiomic features were extracted from regions of interest on T2WI, CE-T1WI, and SUB-T1WI using PyRadiomics.

The extracted features included first-order, shape, gray level co-occurrence matrix (GLCM), gray level run-length matrix (GLRLM), gray level size zone matrix (GLSZM), gray level dependence matrix (GLDM), and neighboring gray tone difference matrix (NGTDM) features. Features were calculated from original images and from wavelet-transformed and Laplacian-of-Gaussian-filtered images. Z-score normalization was incorporated into the model-development pipeline.

### 2.5 Feature selection and model building for classification

Least absolute shrinkage and selection operator (LASSO) regression was used for feature selection (13). Features with non-zero coefficients were retained for subsequent pairwise classification among HER2-negative, HER2-low, and HER2-positive breast cancers. For each pairwise task, patients were allocated using a stratified 8:2 training-test split. This yielded 61 training and 16 test cases for HER2-negative vs. HER2-positive classification, 91 and 23 cases for HER2-low vs. HER2-positive classification, and 98 and 25 cases for HER2-negative vs. HER2-low classification, respectively. To prevent data leakage, all data-dependent preprocessing and model-development procedures were confined to the training set. During five-fold cross-validation, z-score parameters, LASSO feature selection, and hyperparameter grid search were refit using only the fold-specific training partition. The fitted transformations and model were then applied to the corresponding held-out fold. After cross-validation, each pipeline was refit on the full task-specific training set. No test-set information was used for normalization, feature selection, hyperparameter optimization, model fitting, or model selection.

In this study, seven radiomic classification models were developed to predict HER2 expression categories: logistic regression, k-nearest neighbors (KNN), SVM, random forest (RF), AdaBoost, XGBoost, and a weighted ensemble model. The ensemble model aggregated the outputs of six base classifiers using predefined weights to generate the final prediction. The assigned weights were 6 for logistic regression, 5 for KNN, 5 for SVM, 5 for RF, 4 for AdaBoost, and 6 for XGBoost. This approach was intended to integrate information from classifiers with different learning characteristics. Model selection was based exclusively on training-set results. To use one consistent classifier across all three tasks, the mean of the three task-specific training AUCs was calculated for each algorithm. Logistic regression had the highest mean training AUC and was therefore selected as the final classifier before the test set was examined. The fitted logistic regression pipelines were then evaluated once on the corresponding untouched test sets. Test-set discrimination was assessed using AUC with 95% confidence intervals, sensitivity, specificity, precision, F1-score, and accuracy. Calibration curves, Brier scores, expected calibration error (ECE), and decision-curve analysis were also calculated from the logistic regression test predictions. Clinical net benefit was compared with treat-all and treat-none strategies across the threshold probabilities. The complete radiomics workflow, from cohort assembly to model performance assessment, is summarized in Figure 3.

**Figure 3:**
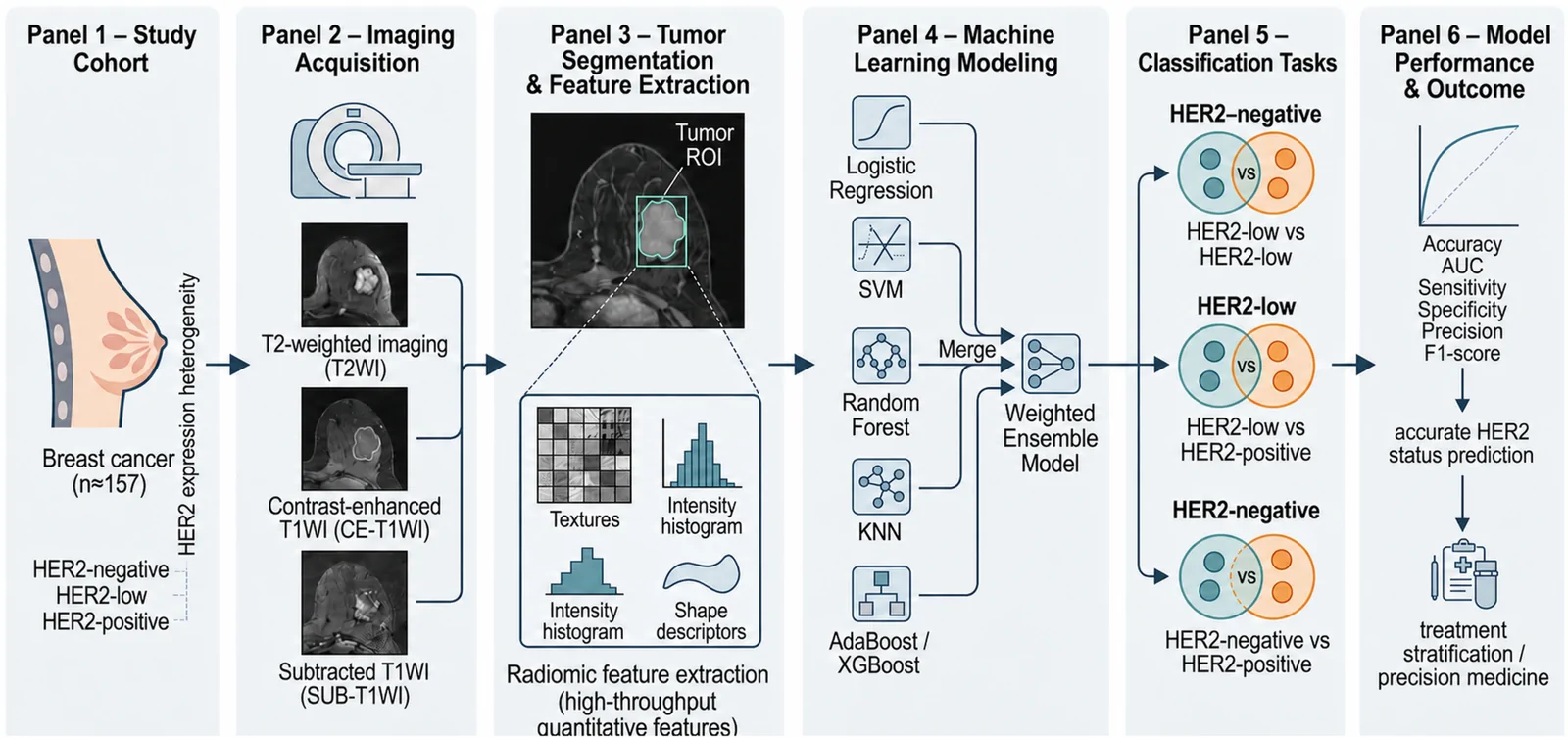
Overview of the multiparametric MRI radiomics workflow for pairwise HER2-status classification. The schematic summarizes cohort assembly (Panel 1), MRI acquisition (Panel 2), tumor segmentation and radiomic feature extraction (Panel 3), candidate classifier development (Panel 4), pairwise HER2 classification tasks (Panel 5), and model performance assessment (Panel 6).

## 3 Results

### 3.1 Training-set feature analysis and model selection

Feature selection and class separation differed across the three tasks (Figure 4). LASSO retained 23 features for HER2-negative vs. HER2-positive classification, 6 for HER2-low vs. HER2-positive classification, and 9 for HER2-negative vs. HER2-low classification. The corresponding mean absolute Cohen’s *d* values were 0.53, 0.70, and 0.22, respectively. The low value for HER2-negative vs. HER2-low classification indicated greater overlap between these groups in the selected radiomic feature space.

**Figure 4:**
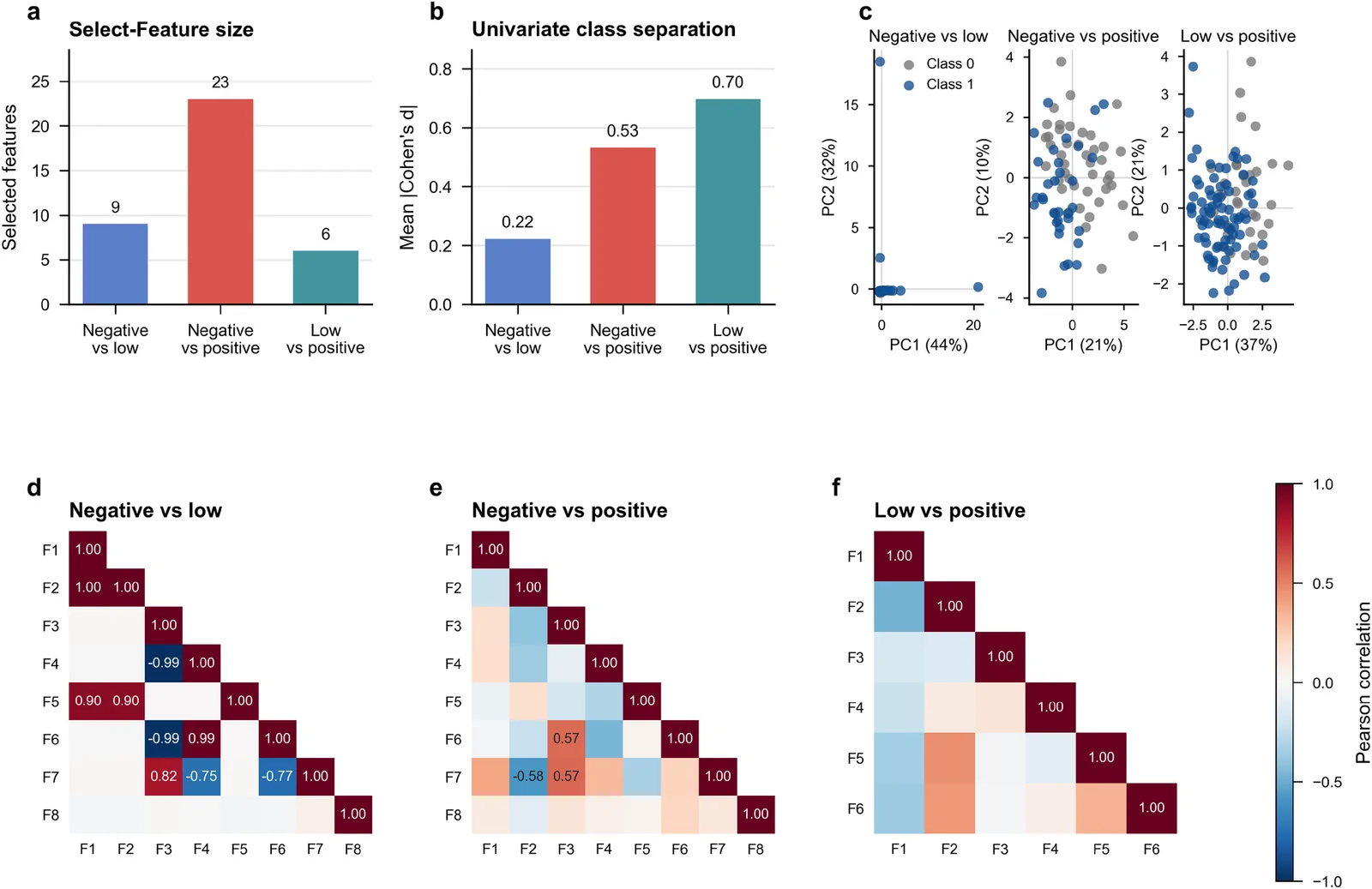
Training-set radiomic feature-space analysis for the three pairwise HER2 classification tasks. **(a)**, Number of features retained after LASSO selection. **(b)**, Mean absolute Cohen’s *d* across the retained features, summarizing univariate class separation. **(c)**, Principal component analysis projections of the selected feature spaces; axis labels report the variance explained by each component. **(d–f)**, Pearson correlation matrices for the displayed selected features in HER2-negative vs. HER2-low, HER2-negative vs. HER2-positive, and HER2-low vs. HER2-positive classification, respectively.

The seven classifiers were compared using training-set performance only (Figure 5). For HER2-negative vs. HER2-positive classification, logistic regression achieved a training AUC of 0.956 (95% CI, 0.902–0.991). For HER2-low vs. HER2-positive classification, its training AUC was 0.774 (95% CI, 0.660–0.875). For HER2-negative vs. HER2-low classification, logistic regression achieved the highest training AUC among the evaluated models, although discrimination remained modest (AUC, 0.604; 95% CI, 0.480–0.729). Across the three tasks, logistic regression had the highest mean training AUC (0.778) and was selected as the common final classifier before test-set evaluation.

**Figure 5:**
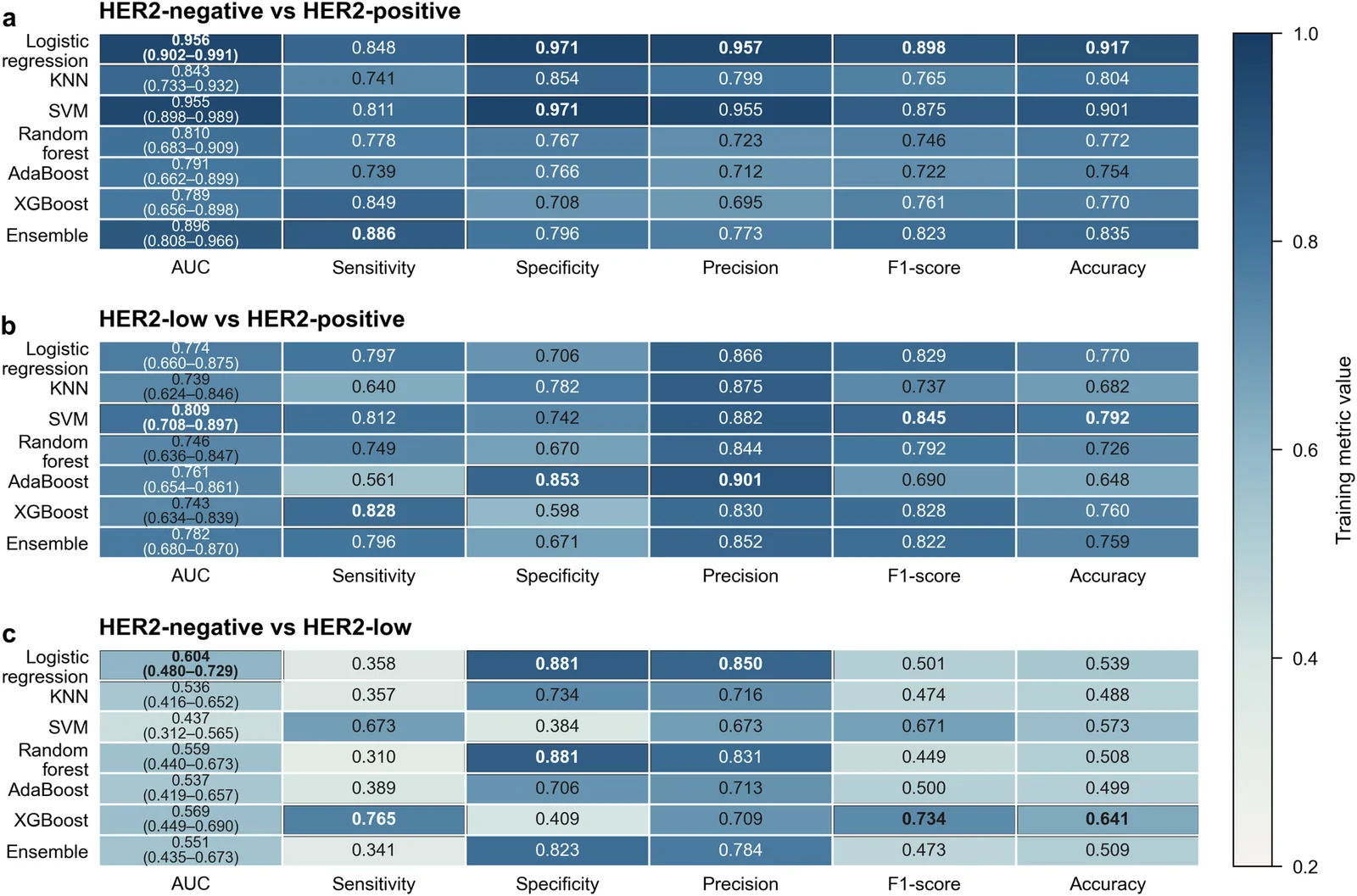
Training-set performance used for classifier selection. Heatmaps compare seven classifiers across three pairwise tasks: HER2-negative vs. HER2-positive **(a)**, HER2-low vs. HER2-positive **(b)**, and HER2-negative vs. HER2-low **(c)**. Rows indicate classifiers, and columns show AUC, sensitivity, specificity, precision, F1-score, and accuracy. AUC values are accompanied by 95% confidence intervals. Bold values with black outlines identify the highest point estimate for each metric within a task. Logistic regression was selected as the common final classifier because it achieved the highest mean training AUC across the three tasks.

### 3.2 Test-set performance of the selected logistic regression models

The three pairwise datasets were divided using a stratified 8:2 training-test split. The HER2-negative vs. HER2-positive task included 61 training cases and 16 test cases; the HER2-low vs. HER2-positive task included 91 and 23 cases, respectively; and the HER2-negative vs. HER2-low task included 98 and 25 cases, respectively. The logistic regression pipelines selected from the training results were evaluated once on these untouched test sets (Table 2; Figure 6).

**Table 2:** Performance of the training-selected logistic regression models on the untouched test sets.

Classification task	Accuracy	AUC	Sensitivity	Specificity	Precision	F1-score
HER2-negative vs. HER2-low	0.520	0.705 (0.493–0.893)	0.245	1.000	0.988	0.382
HER2-negative vs. HER2-positive	0.817	0.922 (0.750–1.000)	0.715	0.894	0.842	0.758
HER2-low vs. HER2-positive	0.737	0.756 (0.529–0.938)	0.746	0.714	0.855	0.791

AUC values are reported with 95% confidence intervals.

**Figure 6:**
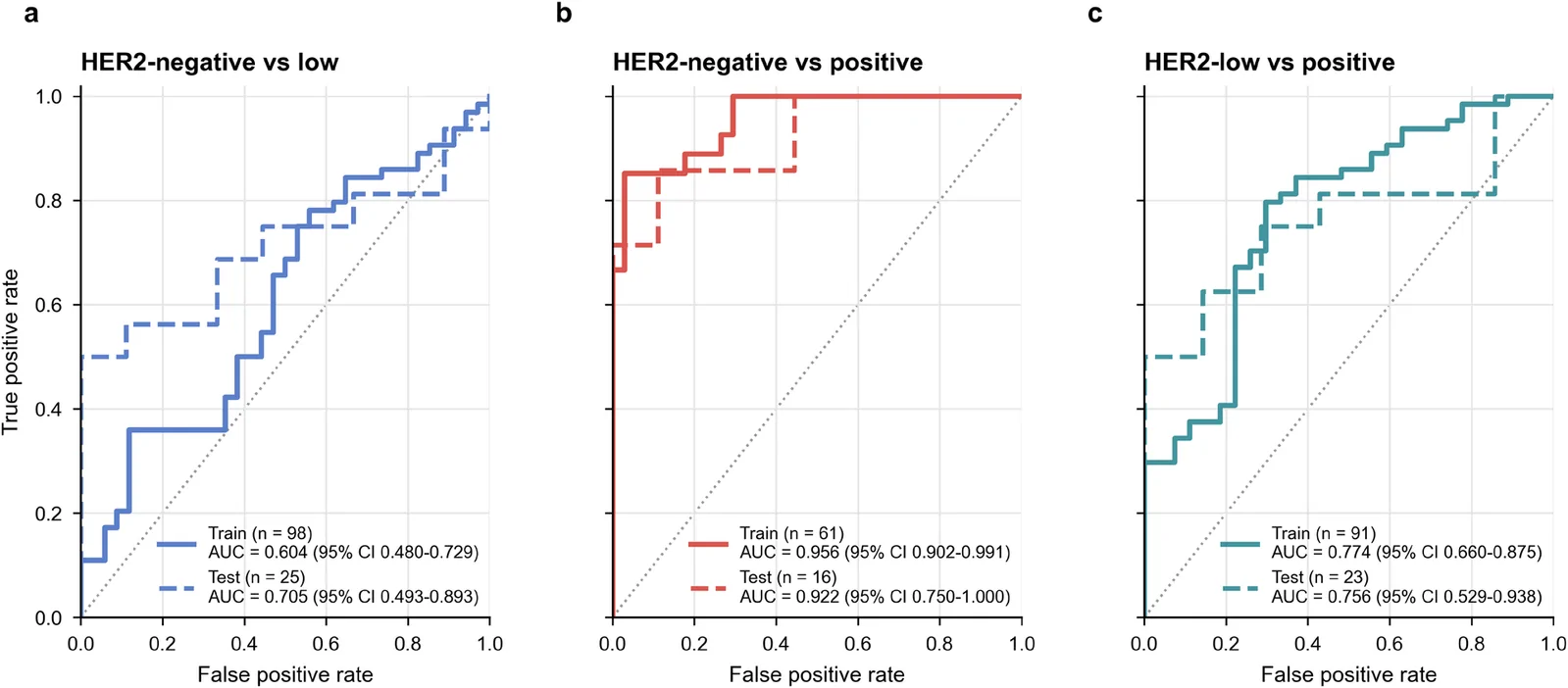
Receiver operating characteristic curves for the training-selected logistic regression models. Panels show HER2-negative vs. HER2-low **(a)**, HER2-negative vs. HER2-positive **(b)**, and HER2-low vs. HER2-positive **(c)** classification.

The strongest test discrimination was observed for HER2-negative vs. HER2-positive classification. The test AUC was 0.922 (95% CI, 0.750–1.000). For HER2-low vs. HER2-positive classification, the test AUC was 0.756 (95% CI, 0.529–0.938). The HER2-negative vs. HER2-low task yielded a test AUC of 0.705 (95% CI, 0.493–0.893), but sensitivity was only 0.245 and accuracy was 0.520. The wide confidence intervals and low sensitivity in this task indicate substantial uncertainty despite the AUC point estimate.

### 3.3 Calibration and decision-curve analysis

Calibration and decision-curve analyses were performed using only the logistic regression test predictions (Figure 7). The HER2-negative vs. HER2-positive model showed the lowest calibration error (Brier score, 0.129; ECE, 0.089). Calibration was less stable for HER2-low vs. HER2-positive classification (Brier score, 0.199; ECE, 0.180) and HER2-negative vs. HER2-low classification (Brier score, 0.234; ECE, 0.201). Decision-curve analysis showed positive net benefit over treat-none across parts of the evaluated threshold range for all three tasks. The HER2-negative vs. HER2-positive and HER2-low vs. HER2-positive models showed benefit across broader ranges, whereas the incremental benefit of the HER2-negative vs. HER2-low model was more limited.

**Figure 7:**
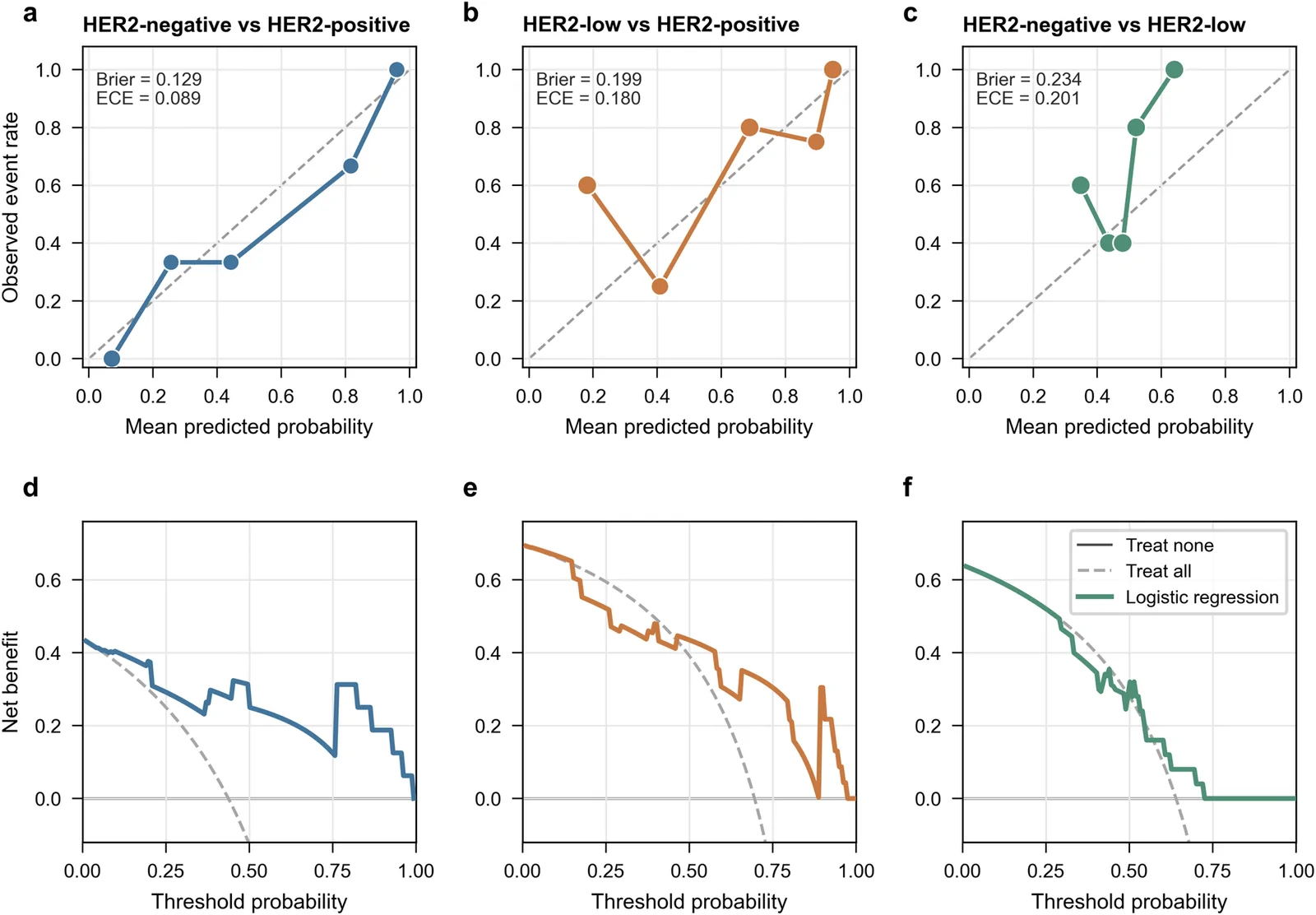
Test-set calibration and decision-curve analysis of the training-selected logistic regression models. **(a–c)**, Calibration plots for HER2-negative vs. HER2-positive **(a)**, HER2-low vs. HER2-positive **(b)**, and HER2-negative vs. HER2-low **(c)** classification. The dashed diagonal indicates perfect calibration; Brier scores and expected calibration errors are shown within each panel. Points denote probability bins, and point size is proportional to the number of test cases in each bin. **(d–f)**, Decision curves for the corresponding tasks. The colored line represents logistic regression model, the solid horizontal line represents treat-none, and the dashed gray line represents treat-all.

## 4 Discussion

In this study, seven multiparametric MRI radiomics classifiers were compared using training data, and logistic regression was selected before the test sets were examined. On the untouched test sets, discrimination was strongest for HER2-negative vs. HER2-positive tumors and moderate for HER2-low vs. HER2-positive tumors. The HER2-negative vs. HER2-low model had an AUC point estimate of 0.705, but its confidence interval included 0.5 and its sensitivity was only 0.245. These findings indicate task-dependent performance and substantial uncertainty when separating HER2-negative from HER2-low disease.

HER2 status has important therapeutic implications in breast cancer. HER2-targeted therapies, including trastuzumab, have established clinical benefit in patients with HER2-positive disease. By contrast, tumors classified as HER2-negative have historically been considered ineligible for such treatment. The recognition of HER2-low breast cancer has challenged this binary framework (14). Emerging HER2-targeted ADCs have further changed the therapeutic landscape for patients previously classified within the HER2-negative group (15). In particular, trastuzumab deruxtecan has shown survival benefit in patients with advanced HER2-low breast cancer compared with chemotherapy (7). These developments highlight the clinical relevance of distinguishing HER2-low tumors from both HER2-positive and HER2-negative disease.

Several recent studies have explored MRI-based radiomics for predicting HER2 status, including the HER2-low category (16–19). Ramtohul et al. reported that an MRI-based radiomic signature achieved AUC values of 0.78 to 0.79 for distinguishing HER2-low plus HER2-positive tumors from HER2-negative tumors (16). Their radiomic signature included six features from T2WI and one feature from CE-T1WI. They also developed a combined model incorporating pathological and MRI features, including histological type, non-mass enhancement, and multiple lesions, for distinguishing HER2-low from HER2-positive tumors. This model achieved AUC values of 0.78 in the training dataset and 0.77 in an external dataset (16). More recently, Hu et al. showed that quantitative dynamic contrast-enhanced MRI parameters could help differentiate HER2-zero, HER2-low, and HER2-positive breast cancers. This finding further supports the premise that contrast-enhanced MRI phenotypes may contain information related to graded HER2 expression (19).

Our study extends this line of work by extracting high-throughput radiomic features from T2WI, CE-T1WI, and SUB-T1WI. A total of 3,369 features were extracted per patient, with 1,123 derived from each sequence. LASSO reduced these feature sets to 23, 6, and 9 features for HER2-negative vs. HER2-positive, HER2-low vs. HER2-positive, and HER2-negative vs. HER2-low classification, respectively. Logistic regression achieved the highest mean training AUC across the three tasks and was selected as the common final classifier. Its test AUCs were 0.922, 0.756, and 0.705 for the three respective comparisons.

The feature-space analysis provided complementary evidence about task difficulty (Figure 4). Mean absolute Cohen’s *d* was lowest for HER2-negative vs. HER2-low classification (0.22), consistent with weak univariate separation. It was higher for HER2-negative vs. HER2-positive (0.53) and HER2-low vs. HER2-positive (0.70) classification. These univariate summaries need not reproduce the ranking of multivariable test AUCs because logistic regression combines information across correlated features.

Inspection of the LASSO-retained feature sets showed task-specific radiomic patterns. For HER2-negative vs. HER2-low classification, the nine retained features comprised one shape, five GLSZM, and three GLCM features, with three features from each MRI sequence. Representative features included major-axis length and zone variance from CE-T1WI; zone variance and large-area low-gray-level emphasis from T2WI; and cluster prominence and informational measure of correlation 2 (Imc2) from SUB-T1WI. These features describe lesion extent, variability in homogeneous-zone size, larger low-gray-level zones, and spatial gray-level dependence.

For HER2-negative vs. HER2-positive classification, all 23 retained features were derived from wavelet- or Laplacian-of-Gaussian-filtered images. The set contained 12 first-order and 11 texture features. Seven features came from T2WI, eight from CE-T1WI, and eight from SUB-T1WI. Representative features included mean, skewness, kurtosis, GLCM correlation, joint entropy, inverse difference moment normalized (Idmn), Imc2, zone variance, and gray-level dependence or run-length emphasis. These multiscale features characterize signal-distribution asymmetry and tail weight, local homogeneity, spatial dependence, and textural randomness. The six-feature HER2-low vs. HER2-positive signature was smaller, comprising four first-order and two texture features. Three features came from CE-T1WI, one from SUB-T1WI, and two from T2WI. It included CE-T1WI Imc2, zone entropy, and kurtosis; SUB-T1WI kurtosis; and T2WI skewness and kurtosis.

Biologically, CE-T1WI and SUB-T1WI texture may reflect heterogeneity in contrast uptake, vascularity, and permeability, whereas T2WI features may reflect extracellular water, edema, necrosis, and stromal organization. Prior multiparametric breast MRI studies linked radiomic texture with enhancement heterogeneity, molecular subtype, and quantitative measures of vascular or cellular heterogeneity (8, 20). The stronger discrimination between HER2-negative and HER2-positive tumors may therefore reflect more distinct multiscale tissue phenotypes. However, LASSO retention denotes predictive selection rather than statistical significance, and filtered radiomic features do not establish independent biological effects or direct radiologic-pathologic correspondence. These interpretations remain hypothesis-generating.

The relatively lower performance for HER2-negative vs. HER2-low classification is clinically and biologically plausible. HER2-low tumors are currently defined by low-level HER2 expression rather than by a distinct imaging phenotype. Their radiomic characteristics may therefore partly overlap with those of HER2-negative tumors. This overlap may explain why the HER2-negative vs. HER2-low task showed lower discriminatory performance than the HER2-negative vs. HER2-positive comparison. Recent breast cancer radiomics studies in other clinically relevant prediction tasks have also emphasized the value of combining imaging features with clinicopathological variables and validating models beyond the development cohort (21, 22). Larger datasets, external validation, and integration with clinicopathological variables may therefore be needed to improve discrimination between these two groups.

This study has several limitations. First, the retrospective single-center cohort was small. The resulting confidence intervals were wide, and performance estimates may be sensitive to the single train-test split. Second, 3,369 candidate features were extracted per patient before selection, creating a substantial dimensionality challenge despite LASSO reduction to 23 or fewer features per task. Third, MRI was acquired on two scanners without scanner harmonization, and the internally estimated calibration and decision curves may be unstable in such small test sets. Fourth, radiomic feature extraction relied on manual tumor segmentation, which is time-consuming and may introduce reader dependent variability. Automated or semi-automated segmentation methods, including deep learning-based approaches, may improve workflow efficiency and reproducibility. Finally, the models lack external validation. Independent multicenter evaluation, scanner harmonization, and prospective assessment are required before clinical translation.

## 5 Conclusions

Among seven classifiers compared using training data, logistic regression achieved the highest mean AUC and was selected for all three pairwise tasks before test-set evaluation. On the test sets, discrimination was strongest between HER2-negative and HER2-positive tumors, whereas HER2-negative vs. HER2-low classification showed low sensitivity and substantial uncertainty. Given the single-center cohort, high initial feature dimensionality, and absence of external validation, these results are strictly exploratory and hypothesis-generating. Independent validation in larger multicenter cohorts using prespecified pipelines is required before clinical application.

## Data Availability

The raw data supporting the conclusions of this article will be made available by the authors, without undue reservation.
